# Retraction: Evaluating the effects of experiencing virtual reality simulation of psychosis on mental illness stigma, empathy, and knowledge in medical students

**DOI:** 10.3389/fpsyt.2026.1904358

**Published:** 2026-06-15

**Authors:** Frontiers Editorial Office

The journal retracts the 17 May 2022 article cited above.

Following publication, concerns were raised regarding the scientific validity of the article. An investigation was conducted in accordance with Frontiers’ policies.

It was found that the complaints were valid and that the article does not meet the standards of editorial and scientific soundness for Frontiers in Psychiatry; therefore, the article has been retracted.

This retraction was approved by the Chief Editors of Frontiers in Psychiatry and the Chief Executive Editor of Frontiers. The authors have not responded to correspondence regarding this retraction.

The authors are invited to submit a revised manuscript once the issues identified have been resolved.

Frontiers would like to thank the concerned readers who contacted us regarding the published article.

